# Supplementary material for: Development of a Low-Cost UV-Vis Spectrophotometer and Its Application for the Detection of Mercuric Ions Assisted by Chemosensors
Source: Sensors (Basel). 2020 Feb 8;20(3):906. doi: 10.3390/s20030906 (PMC7038951; doi:10.3390/s20030906)
Supplement: Supplementary file 1 [file sensors-20-00906-s001.pdf]

Supporting data

Development of a Low-Cost UV-Vis Spectrophotometer and its Application for the Detection of Mercuric Ions Assisted by Chemosensors

David González-Morales [1], Asmilly Valencia [2], Astrid Díaz-Nuñez [3], Marcial Fuentes-Estrada [4], Oswaldo López-Santos [1,*] and Olimpo García-Beltrán [4,*]

[1] Facultad de Ingeniería, Universidad de Ibagué, Carrera 22 Calle 67, Ibagué 730002, Colombia; david.gonzalez@unibague.edu.co

[2] Facultad de Ingeniería Forestal, Universidad del Tolima, Altos de Santa Helena, Ibagué 730001, Colombia; ayvalencial@ut.edu.co

[3] Universidad Nacional de Colombia, Sede Medellín, Escuela de Química, Carrera 65, No. 59A-110, Medellín, Colombia; asdiazn@unal.edu.co

[4] Facultad de Ciencias Naturales y Matemáticas, Universidad de Ibagué, Carrera 22 Calle 67, Ibagué 730002, Colombia; marcial.fuentes@unibague.edu.co

* Correspondence: jose.garcia@unibague.edu.co (O.G.-B.); oswaldo.lopez@unibague.edu.co (O.L.-S.); Tel.: +57-8-2760010

Table of contents

Contents	Page
**Figure S1.** a) 1W used white LED. b) 5° collimator lens.	S3
**Figure S2.** a4988 driver in microstepping configuration.	S3
**Scheme S1.** Synthetic route to 7-(diethylamino)-2 -chromene-2-thione (**3**)	S4
**Figure S3**. ^{1}H NMR (DMSO-d_6) spectrum of 7-(diethylamino)-2*H*-chromen-2-one (**2**)	S5
**Figure S4**. ^{13}C NMR (DMSO-d_6) spectrum of 7-(diethylamino)-2*H*-chromen-2-one (2).	S6
**Figure S5**. ^{1}H NMR (DMSO-d_6) spectrum of 7-(diethylamino)-2*H*-chromene-2-thione (**3**).	S7
**Figure S6**. ^{13}C NMR (DMSO-d_6) spectrum of 7-(diethylamino)-2*H*-chromene-2-thione (**3).**	S8
**Figure S7**. HRMS of 7-(dietilamino)-2*H*-cromeno-2-tiona (**3)**	S9
**Figure S8.** UV-Vis and emission spectra of **3**.	S10
**Figure S9**. (A) UV-Vis spectra of **3** at different concentration; (B) Plot of absorbance of chemosensor **3** against its concentration from **3** to 30 μM.	S11
**Figure S10**. (A) UV-Vis spectra of **3** at different concentration; (B) Plot of absorbance of chemosensor **3** against its concentration from 3 to 30 μM.	S12
**Figure S11.** ^{13}C NMR (DMSO-d_6) spectrum of chemosensor **3** alone (A) and in the presence of mercuric ions (B).	S13

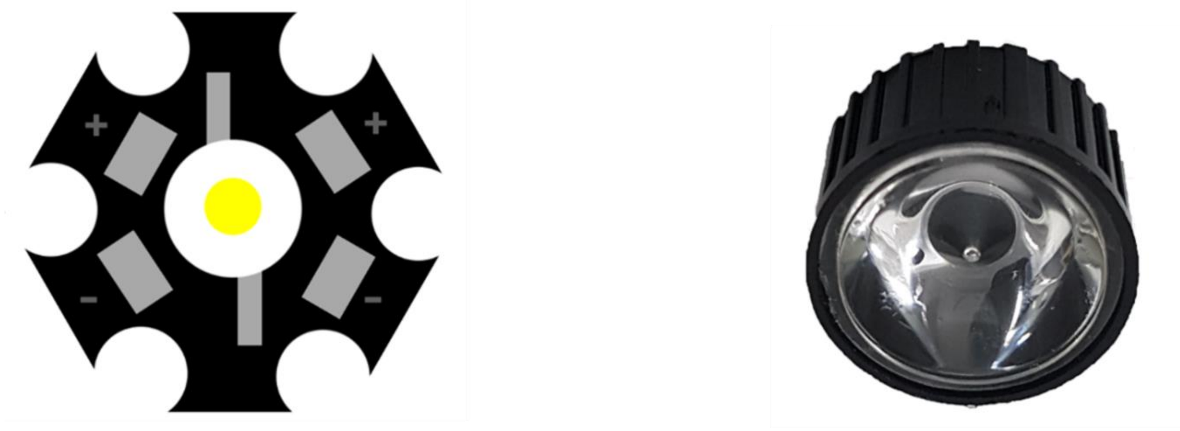

(a) (b)

Figure 1. a) 1W used white LED. b) 5° collimator lens.

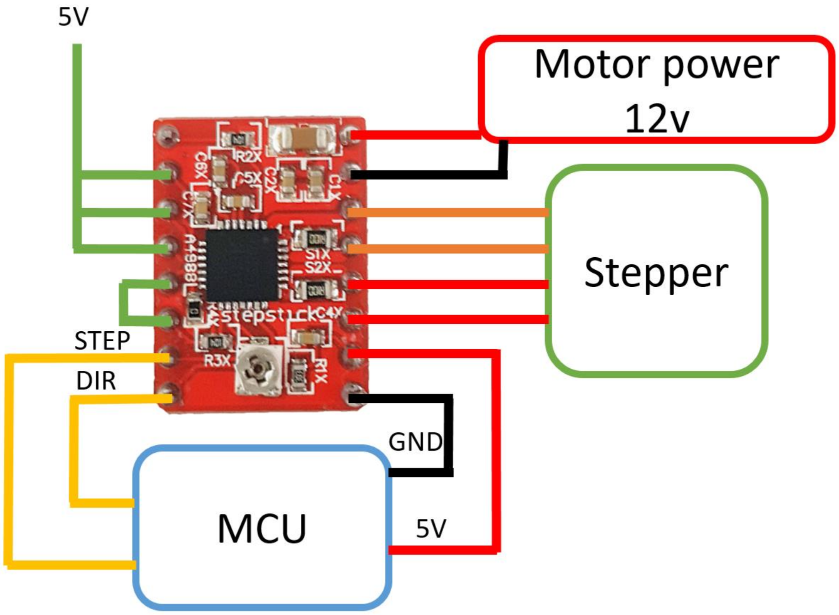

Figure S2. a4988 driver in microstepping configuration.

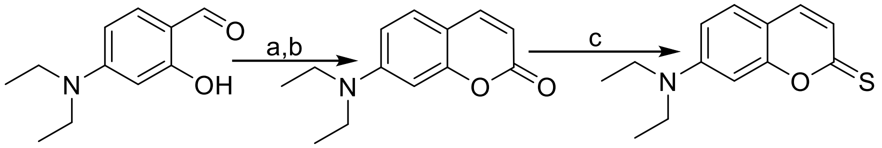

1 **2** **3**

Scheme S1. Synthetic route to 7-(diethylamino)-2 -chromene-2-thione (**3**). Reagents and conditions: a) Diethyl malonate, piperidine, reflux, 6h; b) HCl, AcOH, reflux, 24 h; c) Lawesson Reagent, Toluene, Reflux, 3 h.

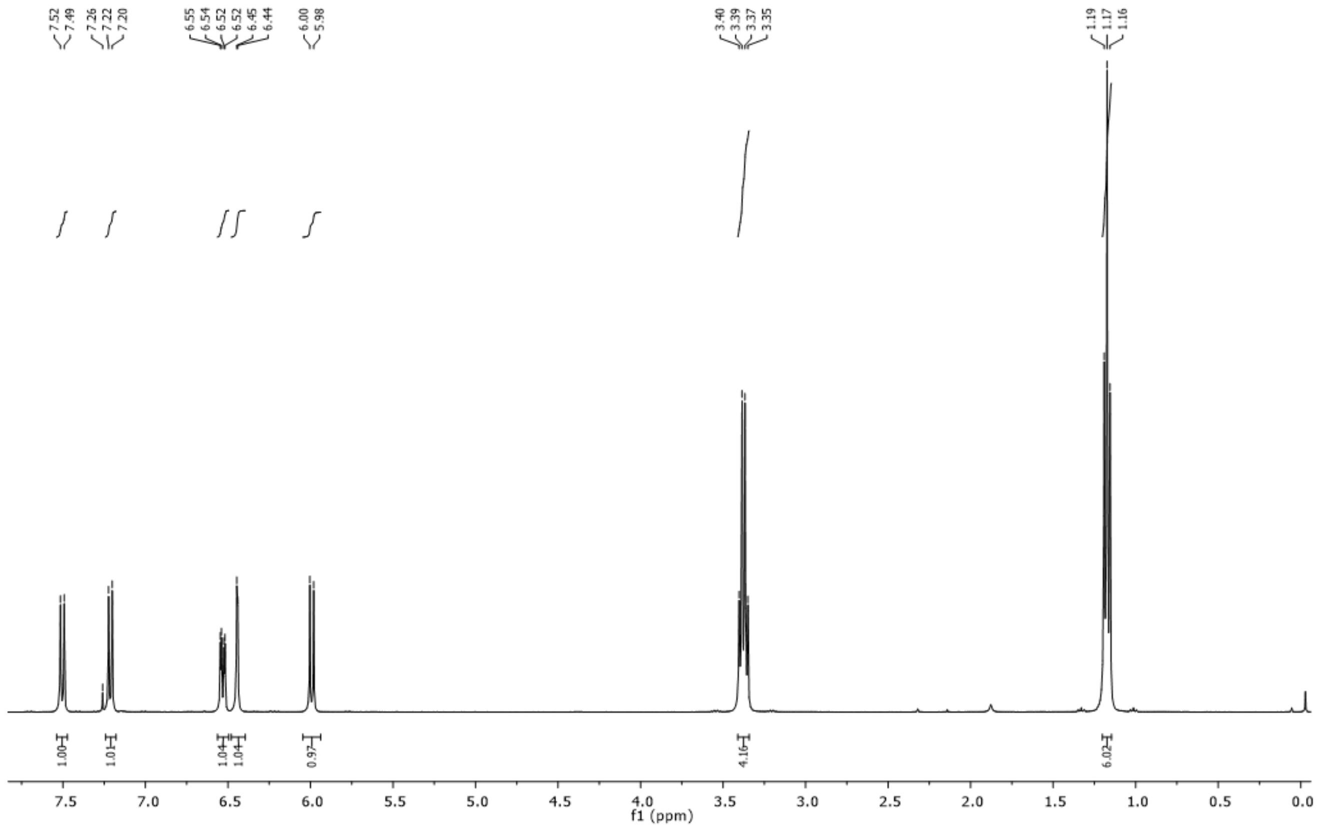

Figure S3. ^{1}H NMR (DMSO-*d*6) spectrum of 7-(diethylamino)-2*H*-chromen-2-one (**2**).

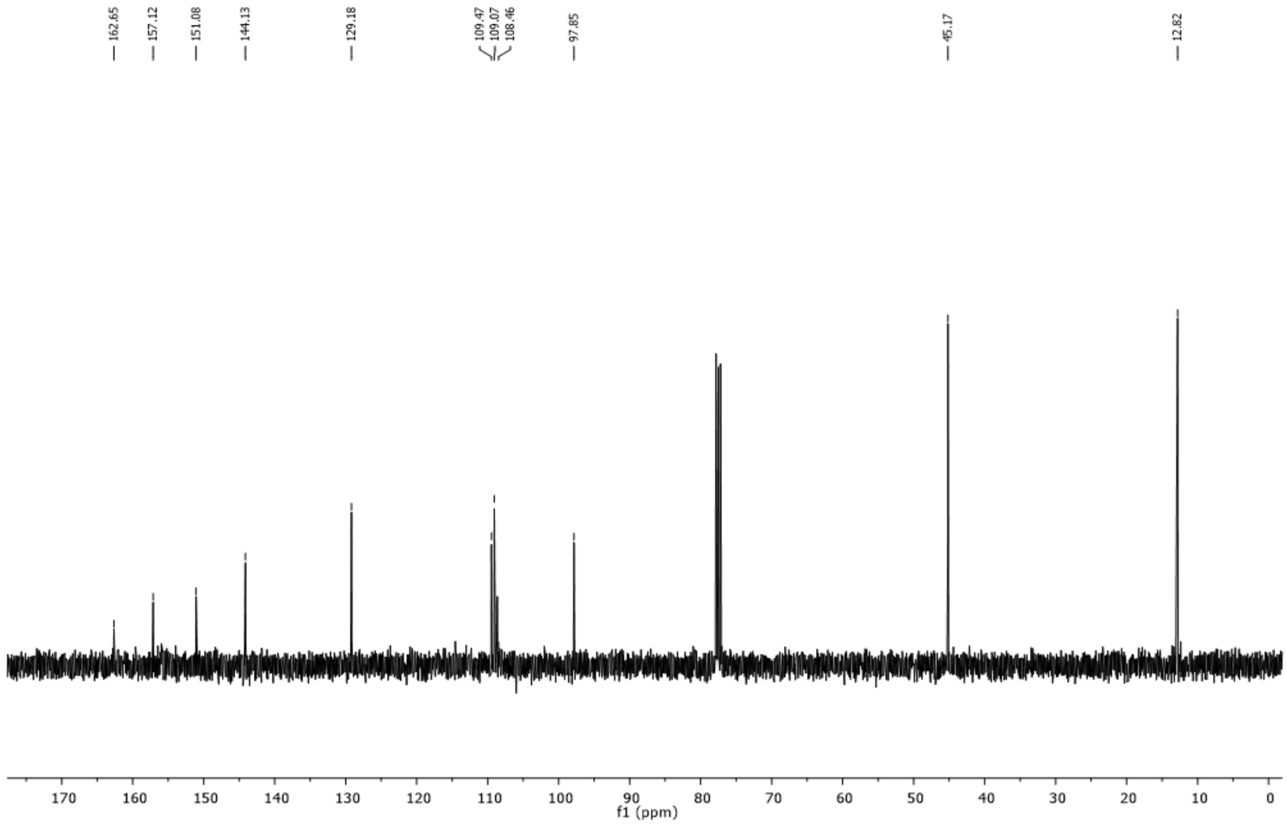

Figure S4. ^{13}C NMR (DMSO-d_6) spectrum of 7-(diethylamino)-2*H*-chromen-2-one (**2**).

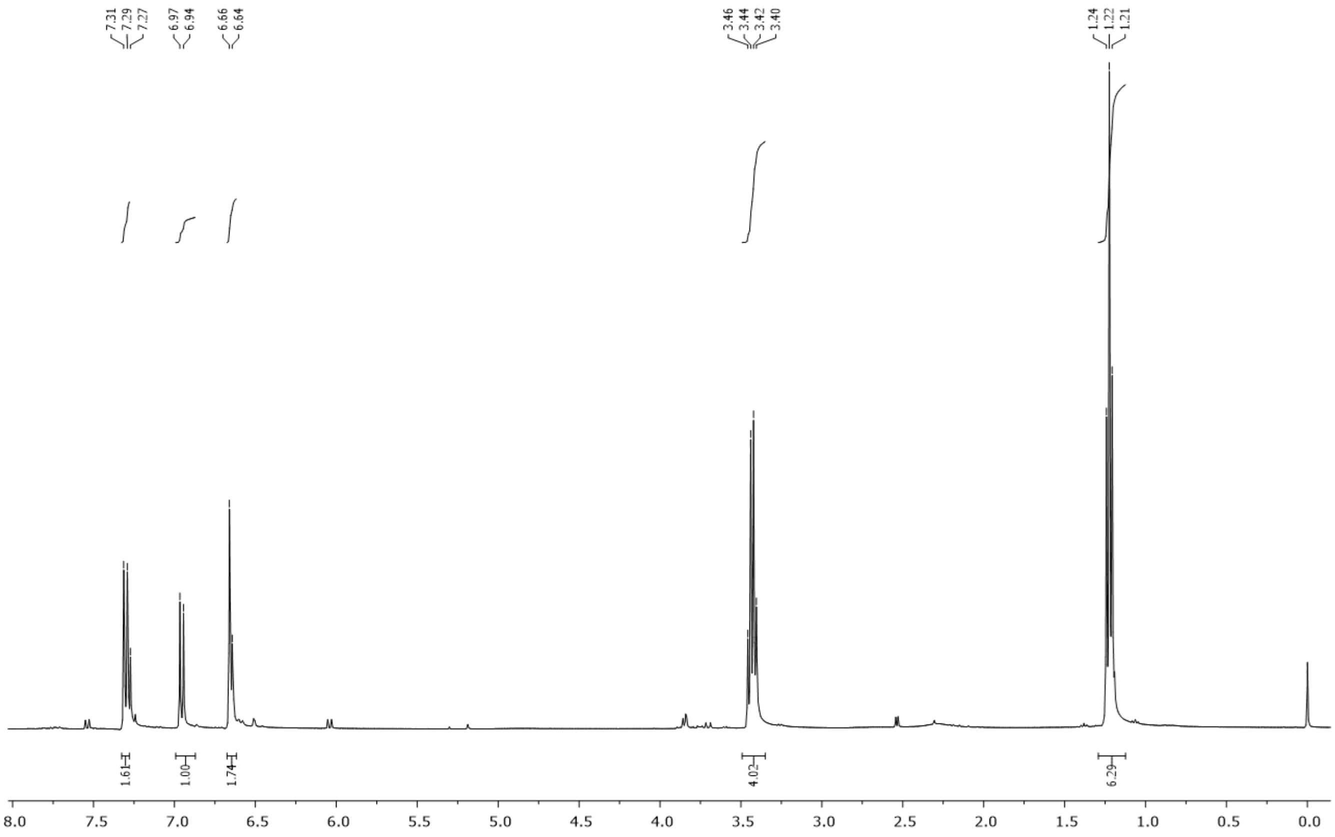

Figure S5. ^{1}H NMR (DMSO-d_6) spectrum of 7-(diethylamino)-2*H*-chromene-2-thione (**3**).

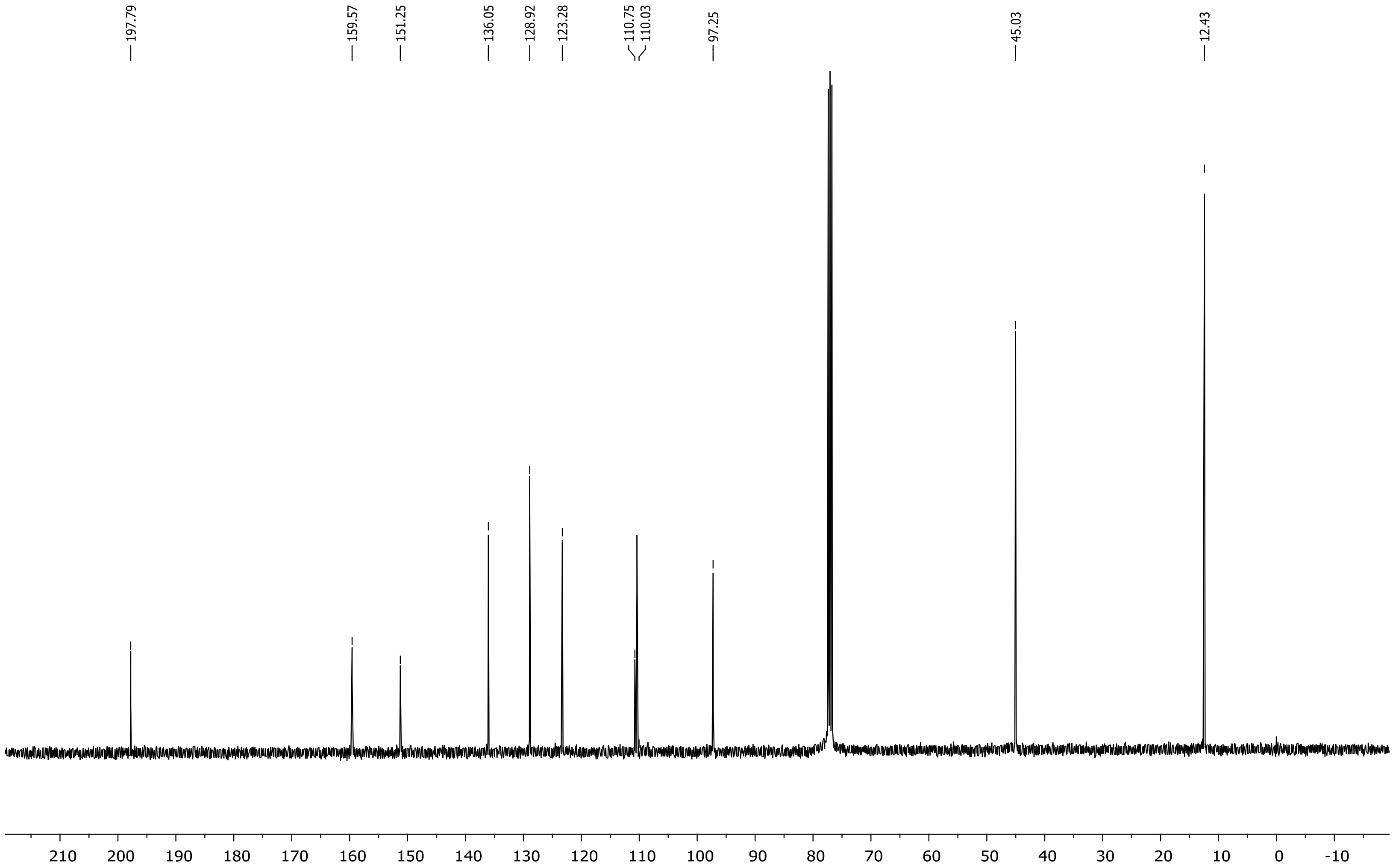

Figure S6. ^{13}C NMR (DMSO-d6) spectrum of 7-(dietilamino)-2*H*-cromeno-2-tiona (**3**).

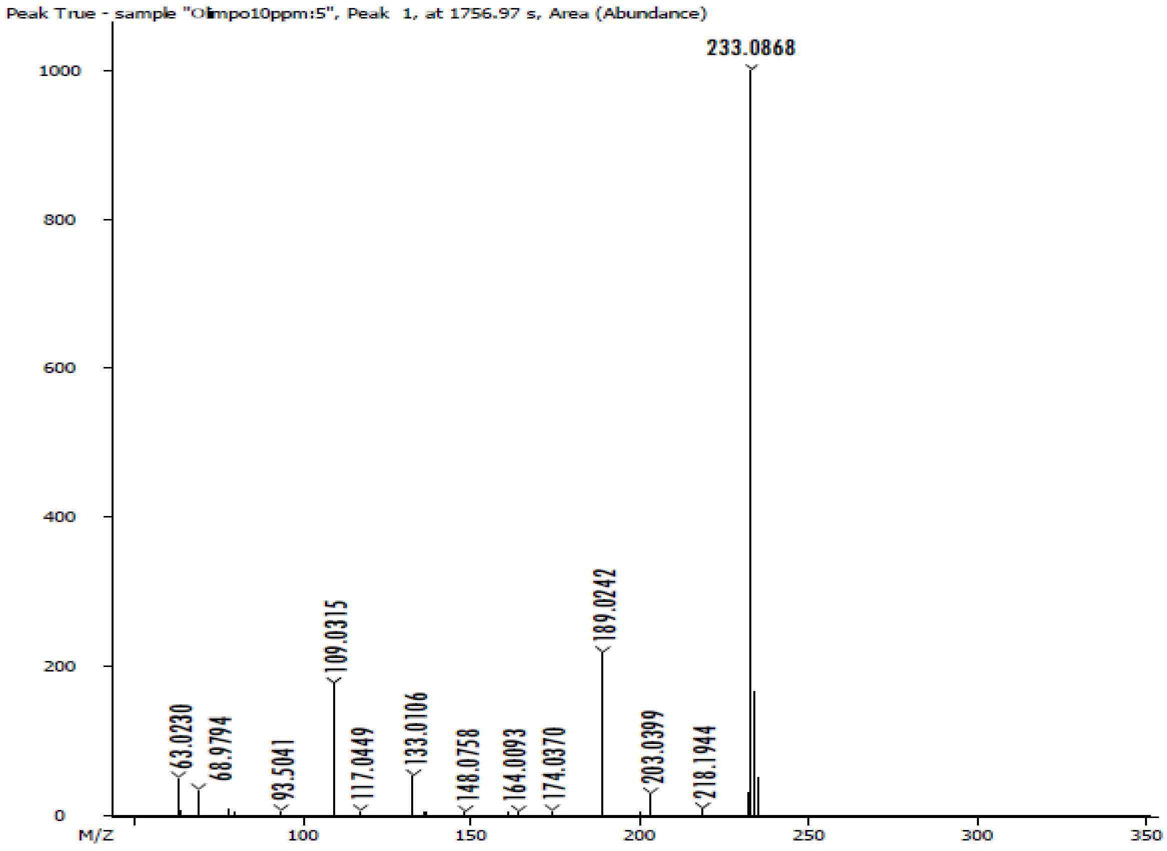

Figure S7. HRMS of 7-(dietilamino)-2*H*-cromeno-2-tiona (**3**).

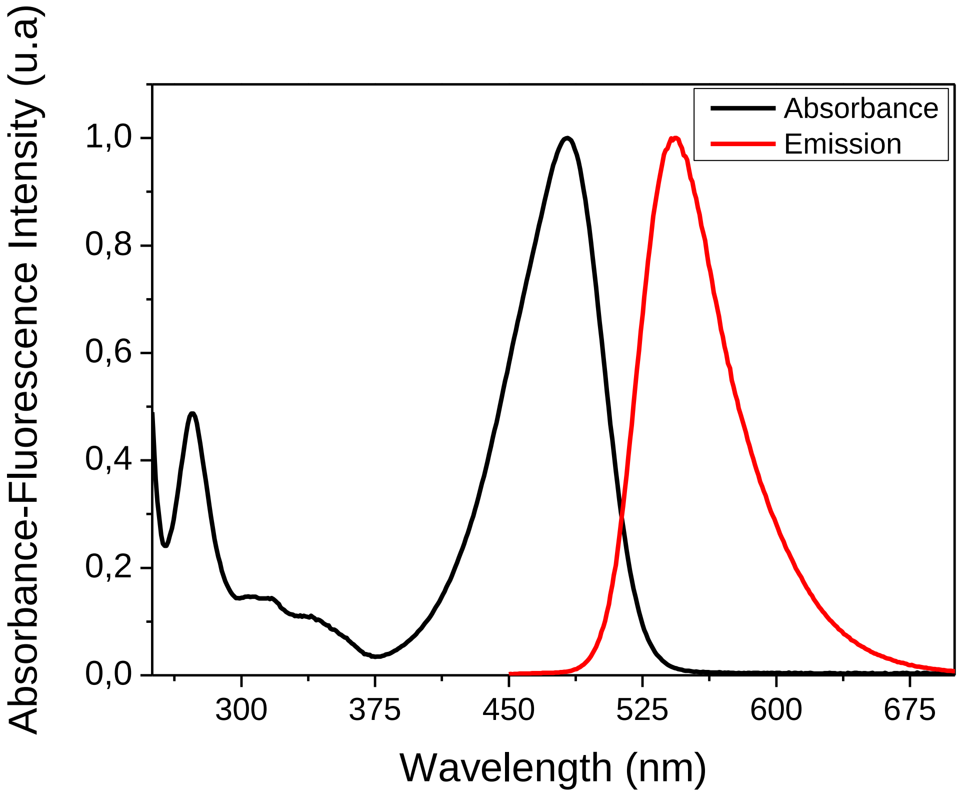

Figure S8. UV-Vis and emission spectra of **3**.

A

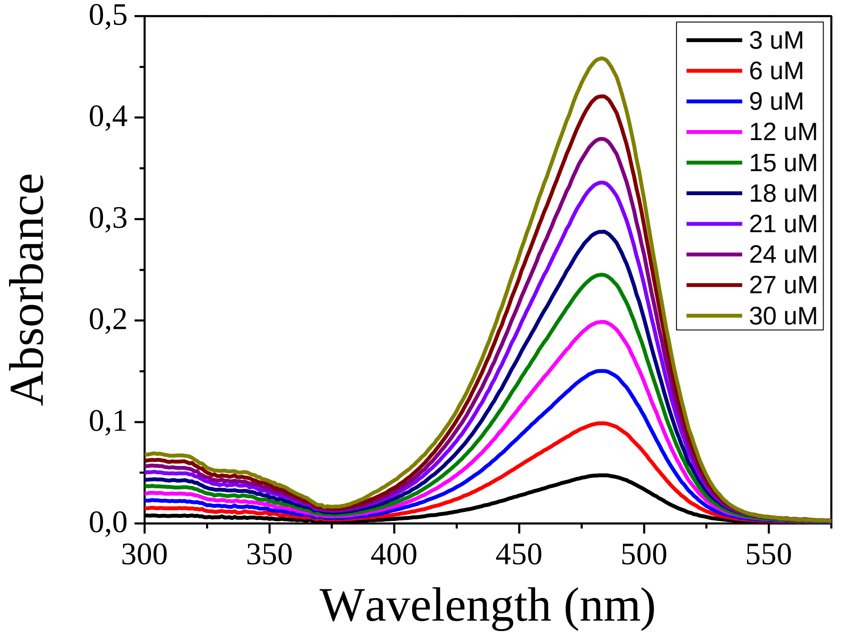

B

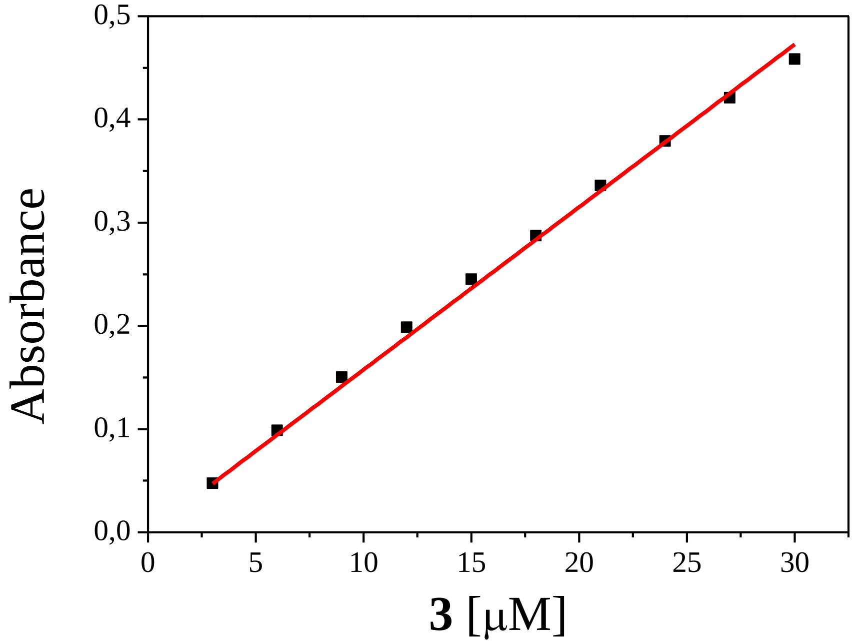

Figure S9. (A) UV-Vis spectra of **3** at different concentration; (B) Plot of absorbance of chemosensor **3** against its concentration from 3 to 30 μM.

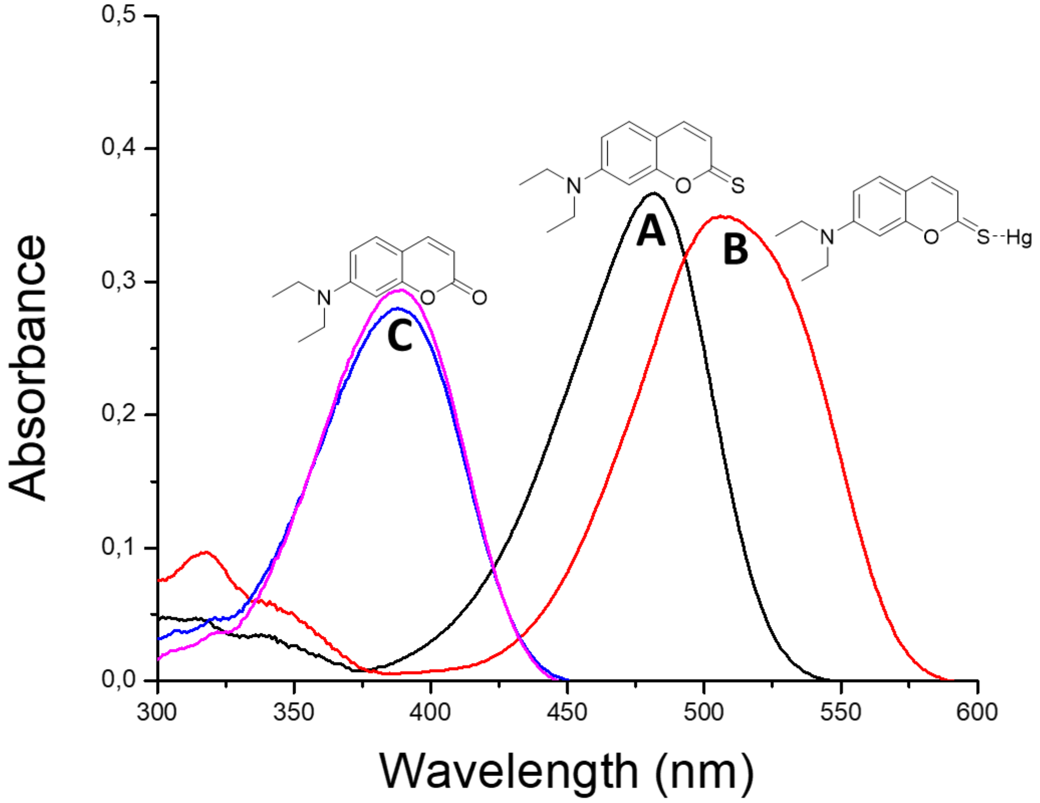

Figure S10. (A) UV-Vis spectra of **3**; (B) UV-Vis spectra of **3**-Hg; (C) UV-Vis spectra of **2.**

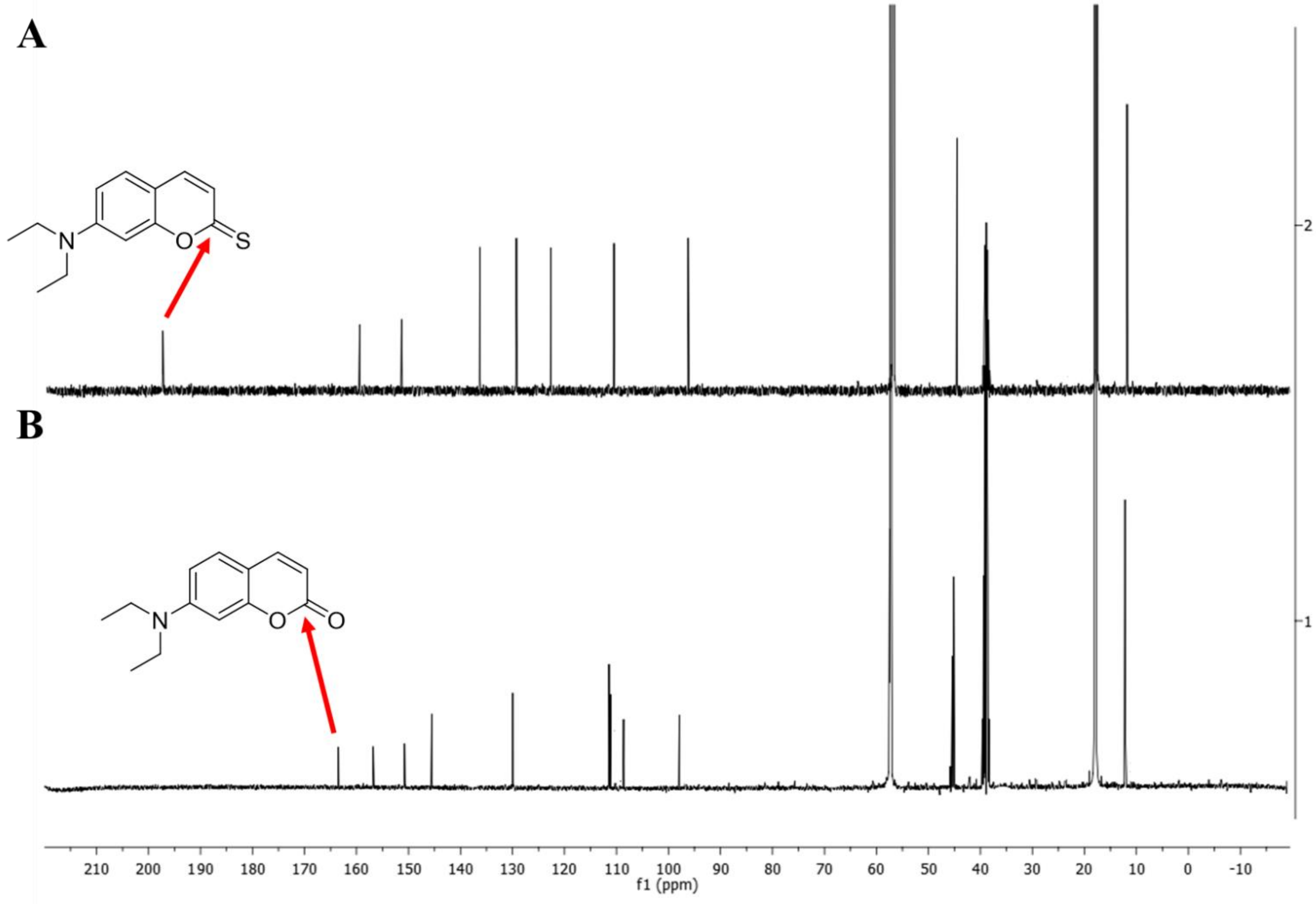

Figure S11. ^{13}C NMR (DMSO-d_6) spectrum of chemosensor **3** alone (**A**) and in the presence of mercuric ions (**B**).
